# Factor structure of the Edinburgh Postnatal Depression Scale in the Japan Environment and Children’s Study

**DOI:** 10.1038/s41598-020-67321-x

**Published:** 2020-07-15

**Authors:** Kenta Matsumura, Kei Hamazaki, Akiko Tsuchida, Haruka Kasamatsu, Hidekuni Inadera, The Japan Environment and Children’s Study (JECS) Group, Michihiro Kamijima, Shin Yamazaki, Yukihiro Ohya, Reiko Kishi, Nobuo Yaegashi, Koichi Hashimoto, Chisato Mori, Shuichi Ito, Zentaro Yamagata, Takeo Nakayama, Hiroyasu Iso, Masayuki Shima, Youichi Kurozawa, Narufumi Suganuma, Koichi Kusuhara, Takahiko Katoh

**Affiliations:** 1 Toyama Regional Center for JECS, University of Toyama, 2630 Sugitani, Toyama, 930-0194 Japan; 2 Department of Public Health, Faculty of Medicine, University of Toyama, Toyama, Japan; 3 Graduate School of Medical Sciences Department of Occupational and Environmental Health, Nagoya City University, 1 Kawasumi, Mizuho-cho, Mizuho-ku, Nagoya, Aichi 467-8601 Japan; 4 National Institute for Environmental Studies, Tsukuba, Japan; 5 National Center for Child Health and Development, Tokyo, Japan; 6 Hokkaido University, Sapporo, Japan; 7 Tohoku University, Sendai, Japan; 8 Fukushima Medical University, Fukushima, Japan; 9 Chiba University, Chiba, Japan; 10 Yokohama City University, Yokohama, Japan; 11 University of Yamanashi, Chuo, Japan; 12 Kyoto University, Kyoto, Japan; 13 Osaka University, Suita, Japan; 14 Hyogo College of Medicine, Nishinomiya, Japan; 15 Tottori University, Yonago, Japan; 16 Kochi University, Nankoku, Japan; 17 University of Occupational and Environmental Health, Kitakyushu, Japan; 18 Kumamoto University, Kumamoto, Japan

**Keywords:** Psychology, Medical research, Epidemiology, Health care, Public health

## Abstract

The Edinburgh Postnatal Depression Scale (EPDS) is frequently used to screen for postpartum depression. However, its factor structure exhibits noticeable inconsistencies between studies. We examined the EPDS at two postpartum time points using a large dataset from outside Western countries. Participants were 91,063 mothers in an ongoing birth cohort of the Japan Environment and Children’s Study. One-, two-, and three-factor structures of the EPDS at 1- and 6-months postpartum were extracted using exploratory factor analysis (EFA) with oblique rotation. Goodness-of-fit indices of extracted factor structures were compared with prior ones by conducting a confirmatory factor analysis (CFA). CFA revealed that a three-factor model extracted from the current EFA—anxiety (items 3, 4, 5, and 6), depression (items 7, 9, and 10), and anhedonia (items 1 and 2)—showed acceptably high goodness-of-fit and invariability across time. These three factors explained about 65% of the total variance with good reliability (all Cronbach’s αs ≥ 0.70). Most three-factor structures (vs. two-) showed higher goodness-of-fit indices. In conclusion, although we only examined the postpartum period, the EPDS likely comprises three dimensions: anxiety, depression, and anhedonia. Our findings raise questions about the one- or two-factor structure of the EPDS.

**Trial registration**: UMIN000030786.

## Introduction

The Edinburgh Postnatal Depression Scale (EPDS) is a questionnaire that was developed to evaluate postpartum depression^1^. Postpartum depression is a common mental illness that can exerts harmful effects on both mothers and their offspring^2,3^. The prevalence of postpartum depressions is approximately 10–15%^4,5^. The EPDS had been validated against clinical diagnosis in over 37 languages since its development^6^, and it has been regarded as the most frequently used and well-validated screening tool for postpartum depression^7,8^.

Although the original purpose of EPDS was postpartum depression screening, recent studies revealed that subscales of the EPDS can be used in new ways, such as anxiety disorder screening^9–11^. Given that postpartum depression is often accompanied by anxiety^3,12^, this means that application of its subscales can help evaluate mothers’ mental health conditions in greater detail^13^. Interestingly, some researchers suggest that there is a difference in pathogenesis between postpartum depression and major depression^14,15^. Thus, identifying subtypes of postpartum depression, such as having anxiety symptoms or not, is also expected to help elucidate the pathogenesis of postpartum depression^16^. Furthermore, recent studies revealed that the subscales predict the lack of mothers’ bonding to their offspring differentially^17,18^. Given that bonding failure might form a hotbed of maltreatment or child abuse^19^, the subscales might inform parental care for beleaguered mothers.

However, despite the common understanding of its actual or potential usefulness, the weakness of EPDS lies in its low factorial validity. That is, whereas a well-designed questionnaire should measure the same construct in various settings, there are many differing views concerning its factor structure. For example, the EPDS was first developed as a one-factor construct^1^. However, studies conducted since its development have suggested two- (e.g. ‘anxiety’ and ‘depression and anhedonia' factor or ‘anxiety and depression’ and ‘anhedonia’ factor^9,20^) or three- (e.g. ‘anxiety’, ‘depression’, and ‘anhedonia’ factor^21^) factor structures. Although no study to our knowledge has indicated a structure of four or more factors, an inconsistent factor structure of EPDS prevents researchers and caregivers from promoting new EPDS applications.

One plausible reason for this discrepancy lies in how the factor structures were extracted. Although there are no gold standard psychometric settings for exploratory factor analysis (EFA), it is recommended (1) not to use principal component analysis (PCA) as an extraction method, (2) not to utilise Kaiser criterion (eigenvalue ≥ 1) for determining the number of factors to be extracted, and (3) not to utilise orthogonal rotation method (i.e. varimax; assuming extracted factors’ correlations to one another are zero) for rotating factors^22^. Additionally, more *N* *is* better when conducting an EFA, for example, *N* ≥ 300 is considered ‘good’ according to Comfrey and Lee^23^, and the goodness-of-fit of an extracted model should be examined via a confirmatory factor analysis (CFA)^22^. Furthermore, it is generally accepted that the factor structure should be assessed at multiple time points. However, to date, scant studies have fulfilled all these criteria simultaneously^21,24,25^, and those that did were all from Western countries.

Therefore, we examined the factor structures of the EPDS utilising a large dataset (*N* > 90,000) from the nationwide birth cohort of the Japan Environment and Children’s Study (JECS). We selected this dataset because it contains EPDS data at two postpartum time points from outside Western countries (i.e. in Japan). To extract factors of the EPDS, an EFA was conducted using settings suitable for psychometrics, using neither PCA, Kaiser criterion, nor orthogonal rotation. Factor structures were obtained, and their goodness-of-fit indices were compared with those derived from previous studies that examined factors with a CFA.

## Results

Data from 91,063 mothers were analysed. Their mean age was 31.3 ± 5.05 years, mean body mass index before pregnancy was 22.5 ± 3.25, 42.6% of mothers were primipara, 36.0% had less than 12 years of education, 40.0% had an annual income of less than 4-million-yen, 4.3% were current smokers, 4.2% were current alcohol drinkers, and 7.9% had a past history of psychiatric illness. The prevalence of postpartum depression—defined by an EPDS score ≥ 9^26,27^—was 14.4% and 11.7% at 1 and 6 months, respectively.

### EFA

The factor structure of the EPDS at 1- and 6-months postpartum, derived from the EFA—with extraction settings for one, two, and three factors—is presented in Table 1. Means and standard deviations of each item are also shown in Table 1. Initial eigenvalues and % cumulative variance (solutions explaining at least ≥ 50% of total variance, which is necessary for meaningful factor analysis^28^) of the first three factors were 4.18 (41.8%), 1.28 (54.6%), and 1.03 (64.9%) at 1 month; and 4.13 (41.3%), 1.33 (54.6%), and 0.997 (64.6%) at 6 months, respectively. The Kaiser–Meyer–Olkin statistic of sampling adequacy at 1 and 6 months were 0.849 and 0.852, respectively (≥ 0.5 suggests appropriateness for factor analysis^29^). Bartlett’s tests of sphericity were significant (*p* < 0.001) both at 1 and 6 months (significance indicates that correlations among items are not constant; thus, they are appropriate for factor analysis).

**Table 1:** Factor structures of the Edinburgh Postnatal Depression Scale derived from exploratory factor analyses using maximum likelihood extraction with Oblimin rotation.

EPDS item		*M* *(SD)*	3-factor				2-factor			1-factor	
			Anx	Dep	Anh	Comm	Anx/Dep	Anh	Comm	Total	Comm
**1 month**^a^											
1^c^	Laugh	0.09 (0.31)	− 0.02	0.03	**0.80**	0.66	0.02	**0.81**	0.67	**0.54**	0.29
2^c^	Enjoyment	0.11 (0.35)	0.01	0.01	**0.85**	0.73	0.06	**0.80**	0.69	**0.57**	0.32
3	Self-blame	1.29 (0.78)	**0.64**	0.01	− 0.04	0.40	**0.67**	− 0.14	0.38	**0.53**	0.28
4	Anxious	1.16 (0.84)	**0.91**	− 0.08	− 0.01	0.75	**0.81**	− 0.11	0.57	**0.66**	0.43
5	Scared	0.51 (0.70)	**0.59**	0.17	− 0.01	0.48	**0.73**	− 0.06	0.49	**0.64**	0.41
6	Hard to cope	1.26 (0.59)	**0.36**	0.00	0.17	0.21	**0.39**	0.11	0.20	**0.44**	0.19
7	Hard to sleep	0.11 (0.40)	− 0.03	**0.68**	0.02	0.45	**0.40**	0.22	0.29	**0.58**	0.33
8	Sad	0.41 (0.59)	0.36	0.43	0.05	0.52	**0.66**	0.11	0.52	**0.73**	0.54
9	Crying	0.12 (0.37)	− 0.05	**0.84**	0.00	0.65	**0.47**	0.24	0.39	**0.66**	0.44
10	Self-harm	0.12 (0.43)	0.08	**0.52**	0.03	0.35	**0.44**	0.16	0.28	**0.56**	0.32
**6 months**^b^											
1^c^	Laugh	0.04 (0.20)	− 0.01	0.05	**0.72**	0.55	0.01	**0.77**	0.59	**0.45**	0.20
2^c^	Enjoyment	0.06 (0.26)	0.01	− 0.02	**0.86**	0.72	0.04	**0.76**	0.60	**0.47**	0.22
3	Self-blame	1.22 (0.81)	**0.64**	0.00	− 0.03	0.40	**0.66**	− 0.12	0.37	**0.55**	0.30
4	Anxious	0.99 (0.81)	**0.89**	− 0.05	− 0.03	0.73	**0.82**	− 0.13	0.59	**0.68**	0.47
5	Scared	0.47 (0.68)	**0.62**	0.13	− 0.03	0.49	**0.73**	− 0.08	0.49	**0.65**	0.42
6	Hard to cope	1.11 (0.57)	**0.38**	− 0.01	0.12	0.19	**0.40**	0.06	0.18	**0.42**	0.17
7	Hard to sleep	0.14 (0.45)	− 0.01	**0.69**	0.01	0.47	**0.45**	0.20	0.33	**0.60**	0.36
8	Sad	0.37 (0.57)	0.35	0.45	0.02	0.54	**0.67**	0.11	0.53	**0.74**	0.55
9	Crying	0.12 (0.37)	− 0.08	**0.84**	0.03	0.65	**0.47**	0.27	0.41	**0.66**	0.43
10	Self-harm	0.16 (0.48)	0.19	**0.46**	0.05	0.38	**0.51**	0.16	0.36	**0.62**	0.39

Bold represents factor loading ≥ .32 on a particular factor, but < .32 on other factors.

*Anx* anxiety, *Dep* depression, *Anh* anhedonia, *Anx/Dep* anxiety and depression, *Comm*. communality.

^a^*n* = 9,038.

^b^*n* = 8,613.

^c^Positively worded item.

In the three-factor solution, extracted factors were identical at 1 and 6 months. The ‘anxiety’ factor consisted of EPDS items 3, 4, 5, and 6; the ‘depression’ factor consisted of EPDS items 7, 9, and 10; and the ‘anhedonia’ factor consisted of EPDS items 1 and 2. Cronbach’s α for ‘anxiety’, ‘depression’, and ‘anhedonia’ were 0.75, 0.72, and 0.81 at 1 month; and 0.74, 0.72, and 0.76 at 6 months, respectively (≥ 0.7 is considered good^30^). Factor correlations between ‘anxiety’ and ‘depression’, ‘depression’ and ‘anhedonia’, and ‘anxiety’ and ‘anhedonia’ were 0.57, 0.54, and 0.42 at 1 month; and 0.60, 0.51, and 0.37 at 6 months, respectively.

In the two-factor solution, extracted factors were also identical at 1 and 6 months. An ‘anxiety and depression’ factor, consisting of EPDS items 3 through 10; and an ‘anhedonia’ factor, consisting of EPDS items 1 and 2, were extracted. Cronbach’s α for ‘anxiety and depression’ and ‘anhedonia’ were 0.81 and 0.81 at 1 month; and 0.82 and 0.76 at 6 months, respectively. Factor correlations between the two factors at 1 and 6 months were 0.50 and 0.45, respectively.

In the one-factor solution, all EPDS items contributed to the one-factor ‘total’. Cronbach’s αs for the total were 0.82 both at 1 and 6 months.

### CFA

**Figure 1:**
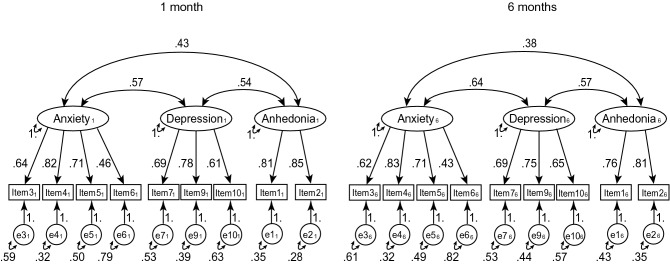
Current three-factor model of the Edinburgh Postnatal Depression Scale, with standardised parameter estimates.

Figure 1 shows a three-factor path diagram that was extracted from the EFA. Table 2 shows various goodness-of-fit indices of the current three-factor model shown in Fig. 1, together with the current two-factor models, and 18 other factor models constructed identically to the one presented in Fig. 1. Goodness-of-fit indices used in the study were χ^2^/degree of freedom (df), adjusted goodness-of-fit index (AGFI), root mean square error of estimation (RMSEA), comparative fit index (CFI), parsimonious CFI (PCFI), Tucker-Lewis index [TLI; also known as the non-normed fit index (NNFI)], standardised root mean square residual (SRMR), and the Akaike information criterion (AIC). As a guideline, smaller χ^2^/df, AGFI ≥ 0.95, RMSEA ≤ 0.06, CFI ≥ 0.95, larger PCFI, TLI ≥ 0.95, SRMR ≤ 0.08, and smaller AIC is considered a good model^31,32^. One model yielded negative error variance on item 10. When comparing one-, two-, and three-factor models, three-factor models yielded better fit than two-factor models, and two-factor models were better than one-factor models, overall. The current three-factor model identified in the EFA yielded acceptable goodness-of-fit; however, the current two-factor models did not attain sufficient fit.

**Table 2:** Goodness-of-fit indices of various factor models of the Edinburgh Postnatal Depression Scale.

Lead author, published year	Country	*N*	Factor model			Goodness-of-fit									
			1	2	3	χ^2^	df	χ^2^/df	AGFI	RMSEA	CFI	PCFI	TLI	SRMR	kAIC
**Previous studies**															
Cox, 1987	UK	63	1–10			122,849	70	1,755	0.774	0.149	0.766	0.596	0.700	0.083	1,834
Berle, 2003	NOR	411													
Gollan, 2017	USA	15,172	1, 2, 6–10			58,964	28	2,106	0.807	0.163	0.813	0.542	0.719	0.075	878
Astbury, 1994	AUS	790	3–5	1, 2, 6–10		83,988	68	1,235	0.845	0.125	0.840	0.635	0.789	0.070	1,795
Matthey, 2008	AUS	238													
Phillips, 2009	AUS	167													
Swalm, 2010, ante- and post-partum	AUS	4,706	3–5	1, 2, 10		18,227	16	1,139	0.908	0.120	0.932	0.497	0.873	0.083	1,118
Bina and Harrington, 2015	ISR	715	3–5	1, 2, 7–10		74,454	52	1,432	0.841	0.134	0.849	0.613	0.791	0.070	1,540
Cunningham, 2015, on admissions	AUS	571	3–5	1–3, 6–10		89,445	68	1,315	0.831	0.129	0.830	0.627	0.775	0.073	1,801
Cunningham, 2015, on discharged	AUS	543	3–6	6–10	1, 2	24,988	64	390	0.943	0.070	0.953	0.677	0.933	0.039	1,736
Chabrol, 2004	FRN	293	3–7	8–10	1, 2	58,560	65	901	0.873	0.107	0.889	0.642	0.846	0.081	1,770
Small, 2007	AUS	1,168	3–7	9, 10	1, 2, 8	103,127	64	1,611	0.797	0.143	0.804	0.572	0.724	0.082	1,815
Bowen, 2008^a^	CAN	402	3–5	10	1, 2, 8	51,766	22	2,353	0.784	0.172	0.851	0.446	0.716	0.095	1,348
Tuohy and McVey, 2008	UK	440	3–5	7–10	1, 2	20,300	48	423	0.945	0.073	0.959	0.639	0.938	0.037	1,486
Kwan, 2015	SGP	920													
King, 2012	USA	169													
Reichenheim, 2011	BRA	811	3–5	7–10	1, 2, 6	38,341	64	599	0.915	0.087	0.927	0.659	0.898	0.069	1,750
Kubota, 2014	JPN	690	3–5	7–9	1, 2	17,987	34	529	0.937	0.082	0.958	0.582	0.932	0.038	1,348
Takehara, 2018	JPN	1,311													
Chiu, 2017	USA	515	3–6	7–9	1, 2	22,573	48	470	0.939	0.077	0.952	0.634	0.927	0.041	1,598
Coates, 2017	UK	12,166	3–6	7–10	1, 2	24,917	64	389	0.945	0.070	0.953	0.677	0.934	0.040	1,736
Pop, 1992	NED	293													
Kozinsky, 2017, antepartum	HUN	2,967	4, 5	8, 9	1, 2	3,592	12	299	0.973	0.061	0.988	0.395	0.971	0.022	909
Kozinsky, 2017, postpartum	HUN	714	4, 5	3, 6, 10	1, 2	10,528	22	479	0.951	0.078	0.965	0.505	0.933	0.039	1,357
Flom, 2018	MEX	628	4–6	3, 7–9	1, 2	43,164	48	899	0.885	0.106	0.907	0.605	0.861	0.059	1,619
**Current study**															
3-factor	JPN	91,063	3–6	7, 9, 10	1, 2	11,145	48	232	0.972	0.054	0.974	0.649	0.961	0.033	1,546
2-factor	JPN	91,063	3–10	1, 2		64,977	68	956	0.853	0.110	0.876	0.662	0.837	0.061	1,776
1-factor	JPN	91,063	1–10			122,849	70	1,755	0.774	0.149	0.766	0.596	0.700	0.083	1,834
Recommended						Lower		Lower	≥ 0.95	≤ 0.06	≥ 0.95	Higher	≥ 0.95	≤ 0.08	Lower

*df* degree of freedom, *AGFI* adjusted goodness-of-fit index, *RMSEA* root mean square error of estimation, *CFI* comparative fit index, *PCFI* parsimonious CFI, *TLI* Tucker–Lewis index, *SRMR* standardised root mean square residual, *kAIC* Akaike information criterion/1,000.

^a^Negative error variance appeared in item 10.

### Additional analysis

The results of the CFA when item 6 and 8 were included/not included are shown in Table 3.

**Table 3:** Goodness-of-fit indices of various factor models of the Edinburgh Postnatal Depression Scale with/without item 6 and 8.

Factor model			Goodness-of-fit									
Anxiety	Depression	Anhedonia	χ^2^	df	χ^2^/df	AGFI	RMSEA	CFI	PCFI	TLI	SRMR	kAIC
3, 4, 5, 6	7, 8, 9, 10	1, 2	24,917	64	389	0.945	0.070	0.953	0.677	0.934	0.040	1,736
3, 4, 5, 6	7, 9, 10	1, 2	11,145	48	232	0.972	0.054	0.974	0.649	0.961	0.033	1,546
3, 4, 5	7, 8, 9, 10	1, 2	20,300	48	423	0.945	0.073	0.959	0.639	0.938	0.037	1,486
3, 4, 5	7, 9, 10	1, 2	6,905	34	203	0.978	0.051	0.983	0.597	0.971	0.027	1,295

*df* degree of freedom, *AGFI* adjusted goodness-of-fit index, *RMSEA* root mean square error of estimation, *CFI* comparative fit index, *PCFI* parsimonious CFI, *TLI* Tucker–Lewis index, *SRMR* standardised root mean square residual, *kAIC* Akaike information criterion/1,000.

### Sensitivity analysis

The results of sensitivity analyses using the complete dataset were not meaningfully different than those calculated using the full information maximum likelihood (FIML) dataset. In addition, the use of ordinary least squares (OLS) and Promax rotation did not produce meaningful differences.

## Discussion

In this study, we extracted the factor structures of the EPDS, at two postpartum time points, using EFA with settings suitable for psychometrics; using neither PCA, Kaiser criterion, nor orthogonal rotation, taking advantage of a large sample size (*N* > 90,000), and then compared their goodness-of-fit indices to those of various one-, two-, and three-factor solutions from previous studies by conducting CFA. The results supported the current three-factor solution derived from the EFA at both time points; specifically, ‘anxiety’ (items 3, 4, 5, and 6), ‘depression’ (items 7, 9, and 10), and ‘anhedonia’ (items 1 and 2). The current model explained about 65% of the total variance and demonstrated acceptable goodness-of-fit indices. Additionally, the model was found to be stable across time, which is consistent with results reported by Coates et al.^21^, who examined two antenatal and two postpartum periods.

In addition to the current model, there are several other models that have been reported with acceptably high goodness-of-fit indices. These models are very similar to the current model. For example, Coates et al.’^21^ three-factor structure [i.e. ‘anxiety’ (items 3, 4, 5, and 6), ‘depression’ (items 7, 8, 9, and 10), and ‘anhedonia’ (items 1 and 2)], would be identical to the current structure if item 8 was removed from the depression factor. Interestingly, the three-factor models by Kozinszky et al.^25^, Tuohy and McVey^33^, Kubota et al.^16^, Takehara et al.^13^, and Chiu et al.^34^ are similar to ours. Although the best CFI, TLI, and SRMR were attained by the three-factor model presented by Kozinszky et al.^25^ [i.e. ‘anxiety’ (items 4 and 5, ‘depression’ (items 8 and 9), and ‘anhedonia’ (items 1 and 2)], this model only adopted six out of 10 items and yielded the worst PCFI among all models examined. Generally, removal of items leads to improved goodness-of-fit, but at a reduction in content validity. The inverse problem is known as the ‘Bandwidth-fidelity dilemma’^35^. Thus, it should be noted that the best goodness-of-fit indices do not always denote the best model, but the most plausible model describing the data. Taken together, we could conclude that the basic factor structure of the EPDS should be three-factor; ‘anxiety’ = items 3, 4, 5, and (6) ‘depression’ = items 7, (8), 9, and (10), and ‘anhedonia’ = items 1 and 2, where items in parentheses represent a low degree of confirmation. These findings raise questions about the one- or two-factor structure of the EPDS.

If the number of extracted factors was determined using the Kaiser criterion in this study, the number of factors at 1 and 6 months would have been three and two, respectively. This is because the third and the fourth eigenvalue at 1 month were 1.03 and 0.72 (data not shown), respectively, whereas the second and the third eigenvalue at 6 months were 1.33 and 0.97, respectively. However, as noted by Osborne^22^, this criterion does not always yield the best result, as goodness-of-fit indices derived from the three-factor solution were far better than those from two-factor solutions. Thus, when using EFA, utilisation of other criteria such as a scree plot, parallel analysis, minimum average partial criteria, and goodness-of-fit indices, in addition to theoretical considerations, is recommended rather than adopting the Kaiser criterion alone^22^.

The factor structure of the EPDS could plausibly depend on culture and/or language. For example, the cut-off value of the EPDS varies among countries and ranges from 9 (e.g. the Japanese version) to 13 (the original version)^36^. Culturally sensitive cut-off points were recommended by the EPDS developers. This difference is plausible owing to cultural variations in the expression of depressive symptoms^37^. For example, Japanese women are typically reluctant to disclose depressive symptoms^38^. Instead, they tend to express emotional problems by referring to physical problems or concern for their child, whereas the EPDS contains items on neither somatic symptoms nor childcare. This tendency is partially owing to the traditional concepts that emotions are considered a weakness of the mind and enduring physical and/or psychological distress is a virtue. However, despite these variations, previous studies from the UK^21^; Australia^24^; Japan^13,16^; and of U.S. African American, Hispanic, and White samples^34^, which used similar EFA methods similar to ours—using neither PCA, Kaiser criterion, nor orthogonal rotation—consistently supported a similar three-factor structure. Thus, to appropriately examine this problem, it is necessary to do so utilising proper methodology.

Interestingly, factor correlations between ‘anxiety’, ‘depression’, and ‘anhedonia’ in the three-factor model were lower than those from previous studies^21,24,39^. Therefore, compared to previous studies, our result shows high discriminant validity; i.e. factors should not correlate too high (≥ 0.85 is considered problematic^40^). Positively worded items in the EPDS are used only in items constituting ‘anhedonia’ factor, which might contribute to separate ‘anhedonia’ factor from ‘depression’ factor^21^. Such a separation from mixed valences of items (and response scales) has been previously reported^41,42^. In fact, all the two-factor models examined in this study^10,39,43^ consist of an independent anxiety factor and a bounded depression/anhedonia factor. Although our data seems to support ‘depression’ and ‘anhedonia’ being distinct factors, further studies examining this point using bifactor models^42,44^ are needed.

When evaluating each EPDS item, three items were notable. First, while item 6 was not problematic in this study, it has been frequently reported as cross-loaded with anxiety and depression factors^10,21,33,39,44–48^. In contrast, item 8 was deleted in the current study owing to cross-loading but has been regarded as a good item in other studies, having discriminatively high factor loadings for depression alone^21^. However, the same cross-loading tendency was observed in Kubota et al.^16^, who also used the Japanese version of the EPDS. Given Coates et al.’^21^ indication that item 6 is open to interpretation, the essence of item 6 may have been altered during translation. In contrast, item 8 might reflect characteristics of Japanese women: unwilling to express their emotions, as previously mentioned. Perhaps this is a difficult question for Japanese women to answer ‘yes’ to. Supporting this view, the raw score of item 8 (approximately 0.4) in our study was in fact far lower than that (approximately 0.8) in a previous study^21^, though measured timing was not the same. Further studies examining this point taking advantage of translated versions of the EPDS are needed.

Second, a previous study conducted by Chiu et al.^34^ excluded item 10 (self-harm) before conducting analyses to avoid negative eigenvalues. They noted that the existence of a ‘quite rare’ response category sometimes yields negative eigenvalues. In fact, the answer ‘quite often’ only occurred 0.8% of the time in that study. However, inclusion of item 10 in the current analyses was not an issue even though the answer ‘quite often’ only occurred among 0.5% of our respondents, which was lower than the 0.8% rate in Chiu et al.^34^. Considering that suicide is a leading cause of maternal death^49^ and self-harm is associated with an increased risk of suicide^50^, the existence of EPDS item 10 is significant in and of itself^51^. While this was an interesting and welcomed attempt to reorganise the EPDS^9,52^, as it will promote a new application of EPDS and will lead to improved performance of EPDS concerning factorial validity, sensitivity, and/or specificity, item 10 should not be excluded merely because it may lead to problematic analyses.

Limitations of the current study include the following: first, we examined the factor structure of the EPDS at two postpartum time points but did not examine factor structure antepartum. Prior studies support both the differences^25^ and similarities^10,21^ between antepartum and postpartum time periods. Further studies should examine this issue. Second, in relation to the above-mentioned point, we did not measure EPDS before 1 month and after 6 months. Third, while our sample consisted of JECS participants, which is a nationwide birth cohort, the extent that our results are generalisable to other populations cannot be determined. For example, it is not clear whether the present factor structure also yields the best good-of-fit indices in fathers.

Despite these limitations, analyses revealed that a three-factor structure consisting of anxiety (items 3, 4, 5, and 6), depression (items 7, 9, and 10), and anhedonia (items 1 and 2) showed acceptably high goodness-of-fit, invariability across postpartum time points, sufficient explanation of total variance, and good internal reliability. The EPDS likely consists of three dimensions: anxiety, depression, and anhedonia. These findings raise questions about the one- or two-factor structure of the EPDS and may shed light on why the EPDS factor structure has been equivocal. We hope that our results will inform new usages of EPDS such as anxiety disorder screening. Further studies examining other populations and/or the antepartum period may prove fruitful.

## Methods

### Participants and design

Participants consisted of mothers taking part in the JECS. The JECS is an ongoing nationwide government-funded birth cohort study of various environmental factors, as well as children’s health and development. Recruitment for the study occurred across 15 regional centres, including both rural and urban locations, throughout Japan, from January 2011 to March 2014. The detailed design and baseline characteristics of the JECS cohort have been published previously^53,54^.

The authors assert that all procedures contributing to this work comply with the ethical standards of the relevant national and institutional committees on research involving human participants and with the Helsinki Declaration of 1975, as revised in 2008. All procedures involving human participants were approved by the Ministry of the Environment’s Institutional Review Board on Epidemiological Studies (no. 100910001), the ethics committees of all participating institutions, and the Ethics Committee of the University of Toyama (no. R2019035). Written informed consent was obtained from all participants.

**Figure 2:**
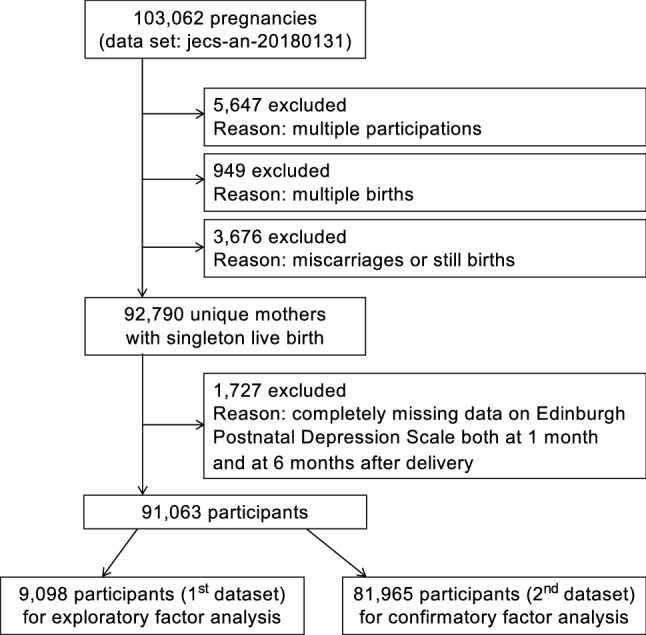
Study flow chart.

This study used the dataset *jecs-an-20180131*, which was released in March 2018 and contains 103,062 pregnancies. To arrive at the number of unique mothers who participated for the first time, 5,647 pregnancies were excluded because of multiple registrations, 949 pregnancies were excluded because of multiple births, and 3,676 pregnancies were excluded because of miscarriage or stillbirth (Fig. 2). Among the remaining 92,790 unique mothers with singleton live births, 1,727 mothers were further excluded because of completely missing data or no response to the EPDS questionnaires administered at either 1- or 6-months postpartum. Thus, data from 91,063 mothers with singleton live births were analysed.

The remaining 91,063 mothers were divided into two datasets at a ratio of 1:9 (Fig. 2). This ratio was chosen to exceed the pre-existing largest number of participants included in the EFA^21,52^. A stratification random sampling technique, setting 15 regional centres as a stratification variable was used. The first dataset (*n* = 9,098) was used to derive factor structures. The second dataset (*n* = 81,965) was used to calculate goodness-of-fit.

### Measures

A self-administered questionnaire, including the Japanese version of the EPDS^26^ (to be described), was administered to mothers on two occasions—1 month and 6 months after delivery. Questionnaires were distributed 1 month after delivery when participants visited the hospital (at which they gave birth) for their 1-month-old’s health check-up. If the questionnaires could not be collected at the time of the visit, they were returned by mail. The questionnaire at 6 months was distributed and collected via mail.

The EPDS^1,6^ is a 10-item self-administered questionnaire, written in a Likert-type format. Briefly, EPDS items consist of (1) laugh, (2) enjoyment, (3) self-blame, (4) anxious, (5) scared, (6) hard to cope, (7) hard to sleep, (8) sad, (9) crying, and (10) self-harm, and (1) and (2) are positively worded item. Participants were asked to mark their level of agreement with the response made on a 4-point response scale, with total scores ranging from 0 to 30.

The Japanese version of the EPDS was developed by Okano et al.^26^ using a back-translation technique and corresponded well with the original version in item valence and response scales; thus, we considered it a validated translated version^55^. It provided good internal reliability (Cronbach’s α = 0.78), test–retest reliability (*r* = 0.92), and an optimal cut-off score of 8/9 screening for clinical diagnosis of depression (75% sensitivity and 93% specificity). The 8/9 cut-off point was also validated in a study by Yamashita et al.^27^ and provided 82% sensitivity and 95% specificity.

### Statistical analyses

#### EFA

EPDS scores at 1- and 6-months postpartum were separately analysed using the first dataset (*n* = 9,098). In this analysis, extraction was set to maximum likelihood (ML) with an Oblimin oblique rotation, which was the same setting as used in a previous study that had a large sample size^21^. Analyses were repeated three times, setting the extracted number of the factors to one, two, and three. Because no study has indicated a structure of four or more factors, we simply examined all the possible numbers of factors by using the brute force method. The subscale rule was items having a factor loading ≥ 0.32 for a particular factor^29^ and < 0.32 for other factors. Missing values were handled using the FIML method. In cases of completely missing data, the participant was removed because even the FIML method cannot treat completely missing data. Owing to this deletion, the final *n*s were 9,038 at 1 month and 8,613 at 6 months.

#### CFA

Factor structures derived from the EFA, together with 18 other factor structures derived from previous studies^1,9,10,13,16,21,24,25,33,34,39,43–45,52,56–64^, were examined using the second dataset (*n* = 81,965). The criteria of literature selection here were whether it is an original study^1^, whether it has *N* ≥ about 300, or whether it overlaps *N* ≥ about 300 study in the final factor structure. Candidate literatures were gathered by conducting snowball search^65^ including references from recent literatures reviewing or examining various factor structures^21,25^ and by using the outputs, further searching more recent literatures citing them using the PubMed. Consequently, the factor structures examined here include one-, two-, and three-factor solutions. Goodness-of-fit was evaluated in terms of χ^2^/df, AGFI, RMSEA, CFI, PCFI, TLI (NNFI), SRMR, and AIC. As previously mentioned, smaller χ^2^/df, AGFI ≥ 0.95, RMSEA ≤ 0.06, CFI ≥ 0.95, larger PCFI, TLI ≥ 0.95, SRMR ≤ 0.08, and smaller AIC represent a good model^31,32^. However, there are many views as to optimal cut-off values for each index^66^. Missing values were handled using the FIML method.

We conducted a multiple-group analysis where each EPDS, at 1- and 6-months postpartum, forms structurally identical independent models. This analysis did not consider time-series measurement to make it possible to directly compare the present findings with previous ones. The final *n*s were 81,401 at 1 month and 77,239 at 6 months.

#### Additional analysis

Because the EFA result on item 6 and 8 were somewhat equivocal, we conducted additional a CFA where item 6 and 8 were included/not included.

#### Sensitivity analysis

Results from the complete case analysis were compared to those from the FIML analysis to assess the differences between the strategies for addressing missing values. In addition, we also used the OLS extraction method and Promax rotation in place of ML and Oblimin rotation, respectively.

All analyses were performed using SAS software (version 9.4; SAS Institute Inc., Cary, NC, USA).

## Data Availability

Raw data are unsuitable for public deposition owing to ethical restrictions and the legal framework of Japan. See https://www.env.go.jp/chemi/ceh/en/index.html for more details. All inquiries about access to data should be sent to: jecs-en@nies.go.jp. The person responsible for handling enquiries sent to this e-mail address is Dr. Shoji F. Nakayama, JECS Programme Office, National Institute for Environmental Studies.
